# University-Industry Collaboration in China and the USA: A Bibliometric Comparison

**DOI:** 10.1371/journal.pone.0165277

**Published:** 2016-11-10

**Authors:** Ping Zhou, Robert Tijssen, Loet Leydesdorff

**Affiliations:** 1 Department of Information Resources Management, School of Public Affairs, Zhejiang University, Hangzhou, Zhejiang, China; 2 Center for Science and Technology Studies (CWTS), Leiden University, Leiden, The Netherlands; 3 Amsterdam School of Communications Research (ASCoR), University of Amsterdam, Amsterdam, The Netherlands; Universidad Veracruzana, MEXICO

## Abstract

In this study, university-industry collaborations in China and the USA are analyzed in terms of co-authored publications indexed in the Web of Science (WoS). Results show a wide gap between China and the USA: Chinese universities are much less active in collaborations with industry in terms of either publication productivity or collaboration intensity. In selecting local and foreign industrial partners, however, more variation exists among Chinese universities than among US universities. The US system is domestically oriented more than that of China. In the USA, the intensity of university-industry collaboration is determined by research quality, whereas in China this is not the case. In both China and the USA, distance is not critical for the establishment of domestic university-industry collaboration. A high correlation is found between productivity indicators including total publications and university-industry co-authored publications. However, the productivity indicators are less correlated with the intensity of university-industry collaboration. Large research universities with strong ties to domestic industry play critical roles in both national publication systems.

## Introduction

Both universities and industry are producers, although their raw materials and output are completely different. With an economy moving from being driven by physical capital to driven by knowledge, the role of universities evolves over time[1]. In the era when knowledge plays a critical role in economic growth, university-industry relations have attracted growing interests of the research community [2–6]. When a government involves as a third player in a research system, the dynamic relations between university, government and industry can be generalized as Triple-Helix relations [7–8].

Why do universities and industry collaborate and how can one make this relation more efficient? The research community has tried to find answers from different perspectives, such as technology transfer and commercialization [9–11], government roles [7,8,12], and individual motives [13–14]. Other studies show that collaborating with industry may improve scientists’ prestige and reputation [15–17].

Relations between scientists’ academic publication and engagement with industry can be positive [18–21]. The results of collaboration are dependent on the researchers’ strategic approaches–the scientific leverage of collaborating with industrial partners would be higher when academics pursue a more proactive strategy and are selective [22]. Funding may play an important role in university-industry collaboration. An inverted U-shaped curve is found between collaboration and publication output: joint publications increase both with public funding amount and its fraction in university-industry collaboration, but only up to a certain point. When the fraction is above 30–40%, the research output declines [23].

Author affiliations of publications indexed in the citation databases such as the Web of Science (WoS) of Thomson Reuters and Scopus of Elsevier make it possible to quantitatively analyze institutional engagement in university-industry collaboration. For example, Tijssen and his colleagues studied publications co-authored by universities and industry (UIC) [24–26], and have generated university-industry research connections (UIRC) data of the world’s top-500 research universities on the basis of the Leiden Ranking available online at http://www.cwts.nl/UIRC2014. Based on the CWTS data, Wong and Singh [27] found a positive effect of university-industry collaboration on the commercialization of university technology.

Using CWTS data of university-industry collaboration, the current study focuses on university-industry relations in China and the USA–the most productive countries in journal publications in the world–in order to explore the differences between these two countries. Compared to the USA, academic involvement in collaborations with industry is far less developed in China than in the USA. During the period 2009–2012, for example, 6.1% of USA publications were output of university-industry collaboration, whereas this was only 2.7% in China. Many factors may cause the large difference, but universities’ proactiveness in collaborating with industry can be an important one [22].

From this perspective, we focus on university-industry co-authored publications in order to answer the following questions: How do the macro-level UIC-based results relate to the systemic differences between the two countries? How do the meso-level UIC-data, at the university level, relate to data on research income/expenditure? Which country is more ‘efficient’ in terms of output and input?

## Data and Methods

The 2014 version of UIRC in the years 2009–2012 available at http://www.cwts.nl/UIRC2014 is used. From UIRC one can obtain publication productivity of university-industry collaboration of the 750 largest research universities in the world that are listed in the CWTS Leiden Ranking 2014 –another data source of the current study. Relevant data are downloaded and further processed so as to serve our research objectives.

The indicators include UIC productivity, UIC intensity, and indexes describing different types of university-industry collaboration. The UIC productivity is defined as the number of publications with both university and industrial addresses, and the UIC intensity (i.e., %UIC) as the percentage of UIC productivity relative to the total number of publications of a university indexed in WoS.

Three types of university-industry collaborations are distinguished according to the physical distance between a university and its industrial partner: UIC Local, UIC Domestic, and UIC Foreign. The UIC Local (%Local) measures the percentage of UIC publications of a university collaborating with industry located within a range of 50 kilometers away from the city center where the university (or its main campus) is located. This indicator may reflect the relative propensity to engage with partners nearby or within the same urban agglomeration. The UIC Domestic (%Domestic) measures the university’s focus on the national industry. The UIC Foreign (%Foreign) is the percentage of UIC collaborating with business enterprises located abroad, reflecting internationalization of a university in its collaborations with industry.

The 2014 version of the UIRC covers 83 universities in China and 166 in the USA. Publications are classified into seven broad fields, namely: life sciences, medical sciences, mathematics/computer sciences/engineering, earth and environmental sciences, natural sciences, cognitive sciences, and the social sciences. Each publication in the Web of Science database is assigned to one of these seven fields by applying a computer algorithm. More details can be found at: https://www.cwts.nl/research/chairs/science-innovation-studies/uirc2014

Not all the 83 Chinese and 166 US universities are active in publishing with industry in all seven broad fields. For example, 26 of the US and 17 of the Chinese universities covered by the Leiden Ranking 2014, do not have UIC papers indexed in the WoS in 2009–2012 in the broad field of mathematics/computer sciences/engineering. In the social sciences, these figures are 21 and 56, respectively. In the medical sciences, US universities are active in collaborations with industry except four universities that do not have UIC papers in the period under study. In China, however, this number is 25—a share of 30% of the 83 Chinese universities, significantly lower than that of USA.

To enhance reliability, we focus on fields that cover enough UIC publications of both Chinese and US universities. Two broad fields, the life and natural sciences, satisfy this condition. Publications in the category “all sciences” reflect overall performance of a country in university-industry collaboration, and thus will also be analyzed. Since the study is based on the 2014 version of UIRC data, Chinese and US universities not yet included in this data cannot be discussed.

Considering that financial factors play a significant role in university-industry collaboration [23], industry-related income and expenditure of universities will be used for a linear regression analysis. We use two sets of data. Data of US universities are harvested from the 2012 and 2013 versions of the database Statistics Access for Technology Transfer (STATT) of the Association of University Technology Managers (AUTM). STATT provides a variety of data on licensing activity and income, startups, funding, staff size, legal fees, patent applications filed, royalties earned, and so on. Academic licensing data of more than 350 universities, research institutions, and teaching hospitals in the USA and Canada are available and are updated annually. For the Chinese universities, we use the 2015 version of technology transfer income of the Best Chinese Universities Ranking in Social Service (BCURSS) (available at http://www.shanghairanking.com/Chinese_Universities_Rankings/Social-Service-Ranking-2015.html), a product of the Center for World-Class Universities of Shanghai Jiao Tong University (CWCU) known for its Academic Ranking of World Universities (ARWU) since 2005. SPSS is used for the statistical analyses.

## Results

**Fig 1:**
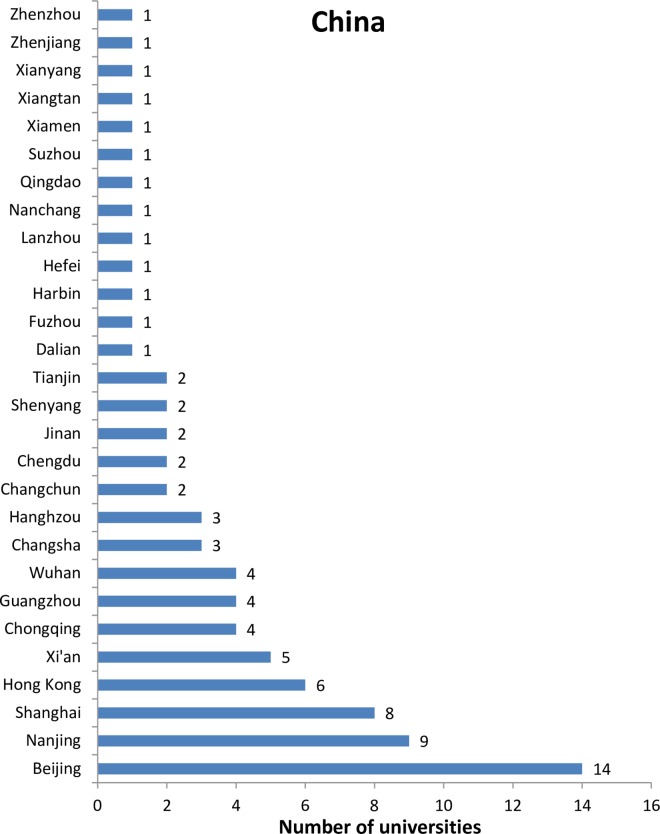
Regional distributions of Chinese universities in the Leiden Ranking 2014.

**Fig 2:**
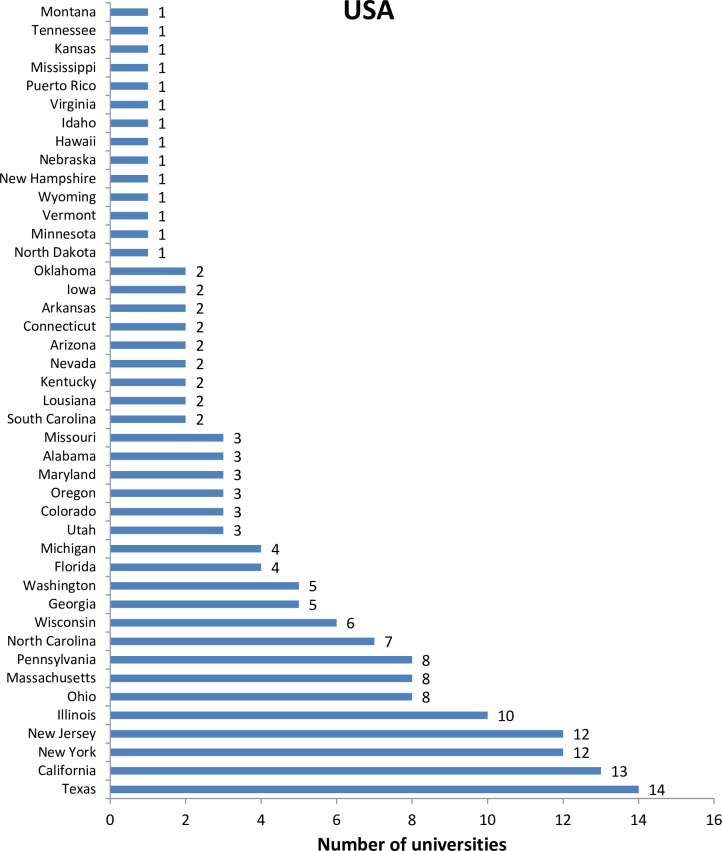
Regional distributions of US universities in the Leiden Ranking 2014.

The 2014 Leiden Ranking covers 750 universities, among which 83 are from China. This is only 3% of the 2,491 higher education institutions of China (National Bureau of Statistics of the People’s Republic of China, 2014. Available at: http://www.stats.gov.cn/tjsj/ndsj/2014/indexch.htm). The low inclusion rate of Chinese universities implies a long way to go for most Chinese universities in terms of publishing internationally, even though China has already been a second largest producer of international publications for some years [28–29]. The regional distribution of universities from both China and the USA included in the Leiden Ranking 2014 are skewed (Figs 1 and 2). Beijing and Texas host the same and the largest number (14) of universities. Compared to the USA, the unevenness is more obvious in China. For example, Beijing hosts five more universities included in the Leiden Ranking than the second region Nanjing, whereas the difference between the first and second largest region in terms of number of universities (i.e., Texas and the California) of USA is only one.

### UIC in all sciences

#### Publication productivity

Among the Chinese universities, Zhejiang University takes the absolute lead, with 1,388 publications more than the second one–Shanghai Jiao Tong University. The two most preferred domestic universities of Chinese students–Peking University and Tsinghua University–publish less than Zhejiang University and Shanghai Jiao Tong University. In the USA, the first position of Harvard University is unshakable, with publication productivity twice as large as that of the University of Michigan at the second position (Table 1).

**Table 1:** Top-10 Universities in Domestic Ranking in publications in “All Sciences” (2009–2012).

Rank	China		P(USA)/P(China)	USA	
	University	P*		P*	University
1	Zhejiang Univ	19,213	2.9	56,018	Harvard Univ
2	Shanghai Jiao Tong Univ	17,825	1.6	28,660	Univ Michigan
3	Peking Univ	17,296	1.6	26,840	Univ Calif—Los Angeles
4	Tsinghua Univ	15,841	1.7	26,768	Univ Washington—Seattle
5	Fudan Univ	13,455	1.9	25,777	Stanford Univ
6	Sun Yat-sen Univ	11,261	2.3	25,715	Johns Hopkins Univ
7	Nanjing Univ	11,067	2.1	23,264	Columbia Univ
8	Sichuan Univ	10,846	2.1	22,599	Univ Calif—Berkeley
9	Univ Hong Kong	10,261	2.2	22,520	Univ Penn
10	Shandong Univ	10,247	2.2	22,182	Univ Calif—San Diego

P* = publications.

Productivity of the leading universities of the USA is significantly higher than that of Chinese universities [30]. The number of publications is at least 1.6 times of those of Chinese universities at the same domestic rank. In fact, productivity of the first publication producer of China, Zhejiang University, is of the same size as that of the 19th producer of the USA: University of California at Davis.

#### University-industry collaboration (UIC)

Not all the Chinese leading (top-10) universities in international publications are active in coauthoring with industry. Of the 10 most productive Chinese universities, four including Sun Yat-sen University, Nanjing University, Sichuan University, and Shandong University are replaced by Chinese University Hong Kong, Peking Union Medical College, Huazhong University of Science and Technology, and Xi’an Jiaotong University when rated in terms of publishing with industry. Compared with the leading Chinese universities, the leading USA universities do better in collaborations with industry. Only two of the leading universities including the University of Pennsylvania and University of California at Berkeley are replaced by Duke University and Columbia University (Tables 1 and 2). UIC productivity of the leading universities of the USA is higher than that of the leading Chinese universities.

**Table 2:** Top-10 Universities in Domestic Ranking in UIC productivity in “All Sciences” (2009–2012).

Rank	China		UIC(USA)/UIC(China)	USA	
	University	UIC(China)		UIC(USA)	University
1	Shanghai Jiao Tong Univ	651	5.8	3756	Harvard Univ
2	Tsinghua Univ	636	3.8	2429	Stanford Univ
3	Zhejiang Univ	547	3.8	2101	Univ Calif—Los Angeles
4	Peking Univ	494	4.0	1998	Univ Washington—Seattle
5	Fudan Univ	442	4.5	1989	Johns Hopkins Univ
6	Univ Hong Kong	302	6.6	1989	Univ Calif—San Diego
7	**Chinese Univ Hong Kong**	291	6.0	1732	Univ Calif—San Francisco
8	**Peking Union Med Coll**	285	6.0	1709	Univ Michigan
9	**Huazhong Univ Sci & Technol**	255	6.6	1691	**Duke Univ**
10	**Xi'an Jiaotong Univ**	251	6.6	1646	**Columbia Univ**

Publications of each of the leading US universities are at least four times that of the leading Chinese universities at the same domestic rank. For example, the largest UIC publication producer of China, Shanghai Jiao Tong University published 651 papers which is only that of the University of Miami (with 653 UIC papers) during 2009–2012 at the 51^st^ position on the list of 166 US universities in the Leiden Ranking 2014.

In terms of UIC intensity measured by percentage of UIC publications in the total publications of a university indexed in the WoS (%UIC), a different list of universities comes to the fore (Table 3). Most of the leading Chinese universities in UIC productivity disappear from the top-10 list of UIC intensity except Tsinghua University, Shanghai Jiao Tong University, and Fudan University (Table 2). In other words, these three universities perform relatively well in both UIC productivity and intensity. Among the leading Chinese universities in UIC intensity, China Pharmaceutical University takes the first position with a UIC intensity of 6.7%, although its UIC productivity is only 134 (Table 3).

**Table 3:** Top-10 Universities in Domestic Ranking in UIC intensity in “All Sciences” (2009–2012).

China			USA		
University	P(UIC)	%UIC	%UIC	P(UIC)	University
China Pharmaceutical University	134	6.7	11.9	339	Rensselaer Polytechnic Institute
China University of Geosciences	160	6.1	10.0	788	University of Maryland, Baltimore
Beijing University of Chemical Technology	148	5.1	9.9	1003	Georgia Institute of Technology
Beijing University of Posts & Telecommunications	76	4.8	9.4	2429	**Stanford University**
Tianjin University	226	4.4	9.3	304	George Mason University
Hong Kong University of Science and Technology	197	4.1	9.0	165	Lehigh University
**Tsinghua University**	**636**	4.0	9.0	852	University of Colorado, Denver
Northeastern University, China	92	3.9	9.0	1989	**University of California, San Diego**
University of Science and Technology Beijing	113	3.7	9.0	518	Carnegie Mellon University
Shanghai University	169	3.7	9.0	355	Thomas Jefferson University
**Shanghai Jiao Tong University**	**651**	3.7	8.8	467	University of Medicine and Dentistry of New Jersey
Zhejiang University of Technology	63	3.3	8.7	1732	**University of California, San Francisco**
**Fudan University**	**442**	3.3	8.5	1549	Massachusetts Institute of Technology
			8.5	276	University of Texas, Dallas
			8.5	660	Indiana University—Purdue University Indianapolis
			8.3	664	University of Texas Southwestern Medical Center, Dallas

In the USA, 44 of the 166 universities included in the Leiden Rankings have a UIC intensity larger than 6.7%, the level of China Pharmaceutical University which ranks first among the Chinese universities. In other words, the UIC intensity in Chinese universities is significantly lower than that of the USA. Similar to the situation of China, most of the American universities leading in UIC productivity no longer appear on the top-10 list of universities in terms of UIC intensity, with the exception of three universities including the University of California San Francisco, University of California San Diego, and Stanford University which perform well in terms of both UIC productivity and intensity (Table 3). Note that these three universities are all located in California.

Using UIC intensity may generate results different from those based on UIC publications. Let us use the 4th university of China (Beijing University of Posts & Telecommunications) in Table 3 as an example: with only 76 UIC papers in four years (2009–2012), the university takes a high position in UIC intensity, which is a distinct contrast to the results based on UIC publications. Similar situations also happen in the case of US universities.

#### Collaboration distance

Most of the Chinese universities leading in UIC publications collaborate more with domestic than foreign industry and with high variation. Not surprisingly, University of Hong Kong and Chinese University Hong Kong have strong ties (>70%) with foreign industry due to specific location and historical background. Peking University also collaborates with foreign industry more than domestically (within mainland China). Nevertheless, the top UIC producer—Shanghai Jiao Tong University—collaborates mostly with domestic industry. Most industrial partners of the leading Chinese universities are located further than 50 kilometers away from the city center where the university (or its main campus) is located (Table 4), which implies less importance of the geographical distance in determining domestic university-industry collaborations in China.

**Table 4:** Collaboration Distance of Top-10 Chinese Universities in UIC Productivity in “All Sciences”*.

Rank	University	P(UIC)	%UIC	%Local	%Domestic	%Foreign
1	Shanghai Jiao Tong Univ	651	3.7	29	76	26
2	Tsinghua Univ	636	4.0	34	55	48
3	Zhejiang Univ	547	2.8	18	54	48
4	Peking Univ	494	2.9	29	44	60
5	Fudan Univ	442	3.3	35	56	51
6	Univ Hong Kong	302	2.9	17	31	71
7	Chinese Univ Hong Kong	291	3.2	13	30	75
8	Peking Union Med Coll	285	3.2	26	54	54
9	Huazhong Univ Sci & Technol	255	2.6	22	60	42
10	Xi'an Jiaotong Univ	251	3.1	25	65	38

* Publications with both domestic and foreign collaborations may result in the percentages not adding up to 100.

In contrast to the leading Chinese universities with different preferences in collaborations with domestic and foreign industry, all of the leading US universities mainly collaborate with domestic industry. Similar to Chinese situation, distance is not critical in establishing domestic university-industry collaborations (Tables 4 and 5).

**Table 5:** Collaboration Distance of Top-10 US Universities in UIC Productivity in “All Sciences”.

Rank	University	P(UIC)	%UIC	%Local	%Domestic	%Foreign
1	Harvard Univ	3756	6.7	26	83	23
2	Stanford Univ	2429	9.4	36	85	21
3	Univ Calif—Los Angeles	2101	7.8	12	85	21
4	Univ Washington—Seattle	1998	7.5	20	88	18
5	Johns Hopkins Univ	1989	7.7	10	83	23
6	Univ Calif—San Diego	1989	9.0	30	85	21
7	Univ Calif—San Francisco	1732	8.7	29	87	21
8	Univ Michigan	1709	6.0	10	84	21
9	Duke Univ	1691	8.2	17	86	20
10	Columbia Univ	1646	7.1	21	86	18

### UIC in the life sciences

#### Publication productivity

With 817 publications more than that of the second largest publication producer Shanghai Jiao Tong University, Zhejiang University takes the absolute lead. Peking University takes the 7th rank whereas Tsinghua University does not appear in the top-10 list. In the USA, the first position of Harvard University is unshakable, with publications twice as many as those of the second one, University of California at Davis. Productivities of the leading universities of the USA are significantly higher than those of Chinese universities. In fact, productivity of the first publication producer of China—Zhejiang University (i.e., 4,363 papers)—is just slightly more than that of the 16th producer of the USA, the University of Pennsylvania (4,275) (Table 6).

**Table 6:** Top-10 Universities in Domestic Ranking in publications in “Life Sciences” (2009–2012).

Rank	China		P(USA)/P(China)	USA	
	University	P*		P*	University
1	Zhejiang Univ	4363	2.8	12249	Harvard Univ
2	Shanghai Jiao Tong Univ	3546	1.8	6344	Univ Calif—Davis
3	China Agr Univ	3359	1.8	6086	Cornell Univ
4	Fudan Univ	3317	1.8	5824	Univ Calif—San Diego
5	Sun Yat-sen Univ	3164	1.8	5753	Univ Florida
6	Peking Union Med Coll	3009	1.9	5586	Univ Washington–Seattle
7	Peking Univ	2930	1.8	5230	Univ Wisconsin–Madison
8	Sichuan Univ	2083	2.5	5149	Johns Hopkins Univ
9	Nanjing Agr Univ	2054	2.4	5028	Univ Michigan
10	Shandong Univ	1956	2.5	4876	Stanford Univ

* P = publications.

#### University-industry collaboration (UIC)

In collaborations with industry in the life sciences, most of the leading publication producers are relatively more active than the rest. Of the leading most productive Chinese universities, only two, namely Nanjing Agriculture University and Shandong University, disappear and are replaced by China Pharmaceutical University and Tsinghua University in the top-10 list of university-industry collaboration (Tables 6 and 7). In the USA, three leading publication producers disappear and are replaced by University of California–San Francisco, Duke University, and University of California–Los Angeles. Huge difference exists between Chinese and US universities in collaborations with industry. Leading US universities are much more active. Compared to the total publication difference between the two countries, the gap in UIC publications is even wider. The leading US universities in the life sciences are much more active in collaborations with industry by producing many more UIC publications than those of China at the same domestic rank (Table 7).

**Table 7:** Top-10 Universities in domestic ranking in UIC productivity in “Life Sciences” (2009–2012).

Rank	China		P(UIC-USA)/P(UIC-China)	USA	
	University	P(UIC)		P(UIC)	University
1	Fudan Univ	113	7.5	844	Harvard Univ
2	Zhejiang Univ	111	3.9	432	Stanford Univ
3	Shanghai Jiao Tong Univ	110	3.9	431	Univ Calif—San Diego
4	China Agr Univ	104	4.0	419	Johns Hopkins Univ
5	Peking Univ	101	3.9	397	**Univ Calif—San Francisco**
6	Peking Union Med Coll	100	3.7	368	Univ Washington—Seattle
7	**China Pharmaceut Univ**	74	4.9	366	**Duke Univ**
8	**Tsinghua Univ**	70	4.9	341	Cornell Univ
9	Sun Yat-sen Univ	67	5.1	340	Univ Florida
10	Sichuan Univ	57	5.8	331	**Univ Calif—Los Angeles**

The UIC intensity of Chinese universities is significantly lower than that of the USA in the life sciences (Table 8). The highest UIC intensity of Chinese universities is 7.8%, whereas that of the US universities is 11.3%. In fact, of the 166 universities included in the Leiden Ranking 2014, 27 have a percentage UIC higher than 7.5%. In terms of UIC intensity, Chinese universities again lag far behind their US counterparts. Of the leading Chinese universities collaborating with industry, four including Tsinghua University, Peking University, Fudan University, and China Pharmaceutical University also take the lead in terms of UIC intensity. In the USA, however, only two of the leading publication producers, namely Stanford University and University of California—San Francisco appear in the top-10 list of UIC intensity (Tables 7 and 8).

**Table 8:** Chinese and US Universities with Top-10 UIC Intensity in “Life Sciences”.

Rank	China			USA		
	University	P(UIC)	%UIC	%UIC	P(UIC)	University
1	China University of Geosciences	9	7.8	11.3	191	Tufts University
2	Zhejiang University of Technology	21	7.4	10.6	52	Northeastern University, USA
3	**China Pharmaceutical University**	74	6.8	9.4	122	University of South Florida, Tampa
4	East China University of Science and Technology	44	5.4	9.4	37	Rensselaer Polytechnic Institute
5	South China University of Technology	38	5.0	9.4	40	Rush University
6	**Tsinghua University**	70	4.8	9.4	173	University of Colorado, Denver
7	Harbin Institute of Technology	20	4.1	9.0	145	Oregon Health & Science University
8	Nanchang University	17	3.7	8.9	432	**Stanford University**
8	Beijing Institute of Technology—BIT	6	3.7	8.8	77	Georgetown University
9	Shanghai University	15	3.6	8.7	13	Boston College
9	Tianjin University	18	3.6	8.4	397	**University of California, San Francisco**
**10**	**Peking University**	101	3.4	8.4	180	University of Utah
10	Nankai University	37	3.4	8.4	36	Loyola University Chicago
10	**Fudan University**	113	3.4	8.4	125	University of Texas Health Science Center, Houston
10				8.3	93	Thomas Jefferson University
10				8.3	129	University of Medicine and Dentistry of New Jersey
10				8.2	78	University of Arkansas, Fayetteville
10				8.2	86	Mississippi State University

#### Collaboration distances

Half or more of the leading Chinese universities collaborate with domestic industry but with some variation. For instance, China Pharmaceutical University mostly (80%) collaborates with domestic industry and some (e.g., Fudan University) have slightly more ties with foreign industry. Most of the industrial partners of the leading Chinese universities in UIC productivity are located farther than 50 kilometers away from the city center where the university (or its main campus) is located. Zhejiang University and Shanghai Jiao Tong University represent two types of collaboration, the former collaborates mostly (80%) with firms more than 50 kilometers away and the later prefers neighboring industry (Table 9). For the leading Chinese universities that are most active in collaborations with firms, distance is not significant in determining university-industry collaboration.

**Table 9:** Collaboration Distance of Top-10 Chinese Universities in UIC Productivity in “Life Sciences”.

Rank	University	P(UIC)	%UIC	%Local	%Domestic	%Foreign
1	Fudan Univ	113	3.4	31	46	58
2	Zhejiang Univ	111	2.5	20	49	55
3	Shanghai Jiao Tong Univ	110	3.1	40	55	48
4	China Agr Univ	104	3.1	37	52	48
5	Peking Univ	101	3.4	33	48	54
6	Peking Union Med Coll	100	3.3	32	57	46
7	China Pharmaceut Univ	74	6.8	34	80	20
8	Tsinghua Univ	70	4.8	34	47	54
9	Sun Yat-sen Univ	67	2.1	21	61	45
10	Sichuan Univ	57	2.7	30	65	37

In the life sciences, domestic collaboration rate of the leading US universities is significantly higher than that of China, and with less variation from the lowest of 73% of Cornell University to the highest of 89% of University of California—San Francisco. Most of the industrial partners of the US universities leading in UIC productivity are located farther than 50 kilometers away from the city center where the university (or its main campus) is located. A large variation, however, exists in this regard. For example, 94% of UIC productivity of Johns Hopkins University are collaborated with non-local firms, whereas Stanford University and University of California–San Diego collaborate relatively more with local industry (Table 10). Similar to Chinese universities, distance is not significant in determining domestic university-industry collaboration.

**Table 10:** Collaboration Distance of Top-10 US Universities in UIC Productivity in “Life Sciences”.

Rank	University	P(UIC)	%UIC	%Local	%Domestic	%Foreign
1	Harvard Univ	844	6.9	32	81	25
2	Stanford Univ	432	8.9	38	88	18
3	Univ Calif—San Diego	431	7.4	38	85	21
4	Johns Hopkins Univ	419	8.1	6	80	26
5	Univ Calif—San Francisco	397	8.4	31	89	16
6	Univ Washington—Seattle	368	6.6	25	86	19
7	Duke Univ	366	7.7	16	78	26
8	Cornell Univ	341	5.6	10	73	34
9	Univ Florida	340	5.9	13	82	20
10	Univ Calif—Los Angeles	331	7.1	10	82	27

### UIC in the natural sciences

#### Publication productivity

In the natural sciences, Tsinghua University is most productive among the Chinese universities. Each of the first five Chinese universities, namely Tsinghua University, Zhejiang University, Peking University, University of Science and Technology of China, and Nanjing University has produced more than 6,000 papers during the period 2009–2012. In the USA, University of California–Berkeley takes the absolutely lead with 8,229 publications, and is the only one with more than 8,000 publications. Productivity of the leading US universities is higher than that of Chinese universities at the same rank except universities at the fifth and sixth positions. Variation of publication productivity of top-10 US universities is higher than that of the Chinese. As the first largest publication producer, University of California–Berkeley has published 3,810 more papers than Princeton University at the 10th position, whereas the publication difference between the first and 10th Chinese universities is 2,554 (Table 11).

**Table 11:** Top-10 Universities in Domestic Ranking in publications in the “Natural Sciences” (2009–2012).

Rank	China		P(USA)/P(China)	USA	
	University	P		P	University
1	Tsinghua Univ	6686	1.2	8229	Univ Calif—Berkeley
2	Zhejiang Univ	6513	1.2	7780	Harvard Univ
3	Peking Univ	6236	1.2	7725	MIT
4	**Univ Sci & Technol China**	6054	1.2	7346	Caltech
5	Nanjing Univ	6029	0.9	5321	Stanford Univ
6	Jilin Univ	5252	1.0	5184	**Univ Michigan**
7	Sichuan Univ	4354	1.1	4828	Univ Calif—Los Angeles
8	**Shandong Univ**	4239	1.1	4774	Univ Maryland—College Park
9	Fudan Univ	4236	1.1	4501	**Univ Wisconsin—Madison**
10	Shanghai Jiao Tong Univ	4132	1.1	4419	Princeton Univ

#### University-industry collaboration (UIC)

Most of the leading Chinese universities are also relatively more active in collaborations with industry in the natural sciences. Of the leading publication producers of China, eight take the lead. The two universities no longer appearing in the top-10 list of university-industry collaboration are University of Science and Technology of China and Shandong University; these are replaced by Tianjin University and Jilin University respectively. The situation is similar in the USA: Two universities, namely University of Wisconsin–Madison and University of Michigan are replaced by University of California—San Diego and Purdue University–Lafayette. Nevertheless, the leading US universities in collaborations with industry produce significantly more UIC papers than those of Chinese universities. Take the first UIC producers of China and the USA, for example, Zhejiang University produced only 182 UIC papers in four years (2009–2012), whereas that of Stanford University was 693. As the 10th UIC paper producer of China, Sichuan University only generated 80 UIC papers, far fewer than that of the 10th UIC paper producer of the USA (i.e., Harvard University) (Table 12).

**Table 12:** Top-10 Universities in Domestic Ranking in Collaborations with Industry in the “Natural Sciences” (2009–2012).

Rank	China		P(UIC-USA)/P(UIC-China)	USA	
	University	P(UIC)		P(UIC)	University
1	Zhejiang Univ	182	3.8	693	Stanford Univ
2	Tsinghua Univ	178	3.2	571	MIT
3	Fudan Univ	154	3.0	466	Univ Calif—Berkeley
4	**Tianjin Univ**	128	3.6	455	Caltech
5	Shanghai Jiao Tong Univ	122	3.6	442	**Univ Calif—San Diego**
6	Peking Univ	117	3.7	436	Princeton Univ
7	**Beijing Univ Chem Technol**	114	3.6	405	Univ Calif—Los Angeles
8	Jilin Univ	82	4.9	398	Univ Maryland—College Park
9	Nanjing Univ	80	4.5	363	**Purdue Univ—Lafayette**
10	Sichuan Univ	80	4.4	353	Harvard Univ

Of the leading Chinese universities in collaborations with industry in terms of publication productivity, only three, namely Beijing University of Chemical Technology, Tianjin University and Fudan University also take the lead in UIC intensity. In the USA, two leading universities, namely Stanford University and University of California—San Diego hold their positions in collaborations with industry in terms of either productivity or intensity. The UIC intensity of the leading Chinese universities is again significantly lower than that of the leading US universities at the same rank, and the situation is even worse than in the life sciences. Take two universities ranked respectively the first in China (i.e., Beijing University of Chemical Technology) and in the USA (i.e., George Mason University) for example, the UIC intensity of the latter is nearly five times of that of the former (Table 13).

**Table 13:** Top-10 Universities in Domestic Ranking in UIC Intensity in the “Natural Sciences”.

China			USA		
University	P(UIC)	%UIC	%UIC	P(UIC)	University
**Beijing Univ Chem Technol**	114	5.3	24.6	153	George Mason Univ
Beijing Univ Posts & Telecom	24	4.7	16.4	23	Univ Texas—Hlth Sci Ctr San Antonio
**Tianjin Univ**	128	4.6	16.1	70	Univ Calif—San Francisco
China Pharmaceut Univ	17	4.4	14.9	63	Univ Maryland—Baltimore
China Univ Geosci	25	4.3	13.0	156	Rensselaer Polytech Inst
Southern Med Univ	6	4.1	13.0	693	**Stanford Univ**
**Fudan Univ**	154	3.6	12.7	86	Lehigh Univ
Second Mil Med Univ	8	3.5	12.4	442	**Univ Calif—San Diego**
Northeastern Univ—China	24	3.4	11.5	16	Baylor Coll Med
China Agr Univ	10	3.2	11.2	27	Univ N Carolina—Charlotte

#### Collaboration distance

Collaboration distance of Chinese universities varies obviously. Of the leading universities most active in collaborations with industry, two including Peking University and Zhejiang University publish more papers with foreign partners than with domestic ones. Most of the UIC papers of Peking University (71%) are collaborated with foreign industrial partners, whereas that of Zhejiang University is 54%. On the contrary, the other eight universities publish more with domestic than with foreign industrial partners, with Beijing University of Chemical Technology and Sichuan University as the extreme. In terms of domestic industrial partners of the leading universities, nine are located further than 50 kilometers away from the city center where the university (or its main campus) is located with Beijing University of Chemical Technology as an exception (Table 14).

**Table 14:** Collaboration Distance of Top-10 Universities of China in UIC Publications in the “Natural Sciences”.

University	P(UIC)	%UIC	%Local	%Domestic	%Foreign
Zhejiang Univ	182	2.8	12	46	54
Tsinghua Univ	178	2.7	29	56	44
Fudan Univ	154	3.6	37	64	44
Tianjin Univ	128	4.6	18	59	42
Shanghai Jiao Tong Univ	122	3.0	29	64	38
Peking Univ	117	1.9	19	29	71
Beijing Univ Chem Technol	114	5.3	67	79	21
Jilin Univ	82	1.6	23	59	43
Nanjing Univ	80	1.3	16	65	36
Sichuan Univ	80	1.8	15	75	29

Similar situation occurs in the leading US universities in the natural sciences. Most university-industry collaboration is domestic and variation in terms of percentage of domestic collaboration among the leading universities is small compared to that among Chinese universities. In terms of distance of domestic collaboration, most industrial partners of the leading US universities are located farther than 50 kilometers away from the city center where the university (or its main campus) is located. Great variation, however, exists in domestic collaboration in terms of distance between a university and its industrial partners: only 4% of UIC papers of Purdue University–College Park are collaborated with local industry, whereas that of University of California—San Diego is 46% (Table 15). Similar to Chinese universities, distance is not significant in determining domestic university-industry collaboration relations.

**Table 15:** Collaboration Distance of Top-10 Universities of the US in UIC productivity in the “Natural Sciences”.

University	P(UIC)	%UIC	%Local	%Domestic	%Foreign
Stanford Univ	693	13	36	83	19
MIT	571	7	20	79	23
Univ Calif—Berkeley	466	6	21	73	29
Caltech	455	6	16	77	26
Univ Calif—San Diego	442	12	46	88	15
Princeton Univ	436	10	18	86	14
Univ Calif—Los Angeles	405	8	23	83	21
Univ Maryland—College Park	398	8	25	88	12
Purdue Univ—Lafayette	363	10	4	87	15
Harvard Univ	353	5	23	79	25

#### Regression analysis

Pearson correlation analysis between publication indicators in the broad fields discussed above (i.e., all sciences, life sciences, and natural sciences) have been investigated. Results show a high correlation between size (or absolute) indicators including total publications, collaborated publications, and publications of university-industry in all areas under investigation. The correlations are more significant in the USA than in China. Correlations between relative indicators (i.e., without size effect) are less significant but stronger than those between absolute (i.e., size-dependent) indicators and relative indicators. A strongly negative correlation is found between relative indicators of domestic and foreign collaboration (r = -0.981; p < .01; see S1A Appendix).

In order to find the drivers of UIC, we performed linear regression with UIC as the dependent variable. Only large research-active universities that satisfy the following conditions are included in this analysis. Firstly, the university should be listed in the Leiden Ranking 2014. Due to data source limitation, we have to use two different sources for the input (funding) data of Chinese and US universities, respectively: data from the Best Chinese Universities Ranking, Social Service Ranking 2015 are for Chinese universities (Table 16), and data from the Statistics Access for Technology Transfer (STATT) of the Association of University Technology Managers (AUTM) are for US universities (Table 17). In total 47 Chinese and 64 US universities (S1B Appendix) satisfy the above conditions and are used in the regression analysis.

**Table 16:** Linear Regression Results of Chinese Universities.

Independent variables	Unstandardized Coefficients		Standardized Coefficients	t	Sig.
	B	Std. Error	Beta		
(Constant)	.030	.006		5.236	.000
Research income from industry (Best Chinese Universities Ranking, Social Service Ranking—2015)	9.484E-10	.000	.021	.225	.823
Total research publication output (CWTS Leiden Ranking 2014)	-4.738E-06	.000	-1.458	-7.080	.000
%UIC Domestic companies (CWTS UIRC 2014)	.000	.000	1.810	8.786	.000
Top 10% cited papers (CWTS Leiden Ranking 2014)	-.051	.067	-.069	-.749	.458

(47 large research-active Chinese universities, dependent variable: %UIC, spurious variables with high pairwise correlations have been removed, R Square (% variance explained) = 0.67).

**Table 17:** Linear Regression Results of US Universities.

Independent variables	Unstandardized Coefficients		Standardized Coefficients	t	Sig.
	B	Std. Error	Beta		
(Constant)	.055	.006		9.041	.000
Industrial research expenditure (AUTM 2012 or 2013)	-3.541E-11	.000	-.111	-1.094	.279
%UIC Domestic companies (CWTS UIRC 2014)	9.118E-05	.000	2.754	6.691	.000
Total research publication output (CWTS Leiden Ranking 2014)	-5.242E-06	.000	-2.588	-6.620	.000
Top 10% cited papers (CWTS Leiden Ranking 2014)	.084	.044	.219	1.911	.061

(64 large research-active Chinese universities, dependent variable: %UIC, spurious variables with high pairwise correlations have been removed, R Square (% variance explained) = 0.54).

In both countries, the research size of a university and its links with domestic companies appear to be the main determinants of UIC intensity. The volume of research funding flows from industry appears to be less relevant. This is similar to the results of Banal-Estañol et al. (2015) on the role of public funding: UIC publications increase with both public funding and the fraction of public fund in university-industry collaboration, but only up to a certain point: with more than 30–40% public funding, research output declines.

A significant difference between the Chinese and US universities is found with regards to the research quality variable; that is, the percentage of research papers in the world’s top 10% most highly cited. The results suggest that UIC intensity is also determined by research quality determinants in the USA but not in China. This implies that strong research ties with industry is concentrated in US universities with high quality research across the board. Nevertheless, the ‘concentration effect does not occur in the case of these 47 Chinese universities. Furthermore, the Chinese research system is more financially driven than in the USA.

## Discussion and Conclusion

Universities most productive in academic publishing are not by definition the most active ones in collaborations with industry. However, strong positive correlations were found between these two factors. Publication productivity correlates highly with research collaboration including university-industry collaboration, but does not necessarily result in high UIC intensity. Universities with high publication productivities may have low UIC intensity even though their UIC productivity is high, and on the contrary, those with low numbers of publications may have high UIC intensity even though their UIC productivity is lower than those of large producers due to size effects. The large research universities with strong ties to industry tend to have high UIC intensity rates. In a national research system, large research universities with strong links to domestic industry play critical roles.

Publication productivities of most leading US universities are significantly higher than those of Chinese universities at the same domestic ranks. This difference is more pronounced in “all sciences” than in the “life sciences”, and less so in the “natural sciences”. US universities are much more active in collaborating with industry than their Chinese counterparts, implying more involvement in the national research system of US universities. Field variation exists in this regards: the distance between Chinese and US universities in collaborations with industry is narrower in the “natural sciences” than in the “life sciences”. In other words, Chinese universities are relatively more active in knowledge transfer in the “natural sciences” than in the “life sciences”.

An important difference is also found between Chinese and US universities in selecting industrial partners: the US system is nationally oriented, whereas the Chinese system is oriented both nationally and internationally. Some Chinese universities prefer domestic industry and some are more involved with foreign industry. Strongly negative correlations between domestic and foreign collaboration were found. A university focusing more on collaborating with foreign industry may be less vigorous in establishing domestic partnership, and vice versa. The national orientation of university-industry collaboration in the USA may imply that the US research system is more self-contained than that of China. In other words, the Chinese research system is perhaps more open than that of the USA.

Another significant difference is found between the two countries: UIC intensity is partially determined by research quality determinants in the USA but not in China. In other words, strong research ties with industry is concentrated in US universities with high quality research environments, but this is not the case in China. The Chinese UIC is more financially driven than that of the USA where it seems more a consequence of academic publication.

The UIC data of the Leiden Ranking enables quantitative studies of university-industry collaboration in terms of academic publication activities at both the macro- and meso-levels for countries and regions, as well as individual research institutions. Most of the indicators are effective and can be used independently except UIC intensity: Universities with high UIC intensity are not necessarily active in collaborating with industry, vice versa. Nonetheless, when used together with UIC productivity UIC intensity still has its value: A university with high value on both indicators would be more active in collaborating with industry than those with only high value of productivity.

The regression analysis is based on publication data of “all sciences”, which cannot sufficiently reflect field variations. University-industry collaborations happen more in the natural and life sciences than in the social sciences. Even in the same field, cost versus output of different UIC projects may vary significantly. Expenditure/income data used in the current study are from two different sources, which may affect data consistency. As patents and publications are important output of university-industry collaboration, the conclusion of the current paper may not reflect the complete picture especially in fields like medical sciences, computer science and engineering in which patents are a major part of the output.

## Supporting Information

S1 Appendix Appendix. (DOCX)
