# Supplementary material for: University-Industry Collaboration in China and the USA: A Bibliometric Comparison
Source: PLoS One. 2016 Nov 10;11(11):e0165277. doi: 10.1371/journal.pone.0165277 (PMC5104362; doi:10.1371/journal.pone.0165277)
Supplement: S1 Appendix — (DOCX) [file pone.0165277.s001.docx]

Appendix

*A. Pearson correlation coefficients (samples: 83 in China, 166 in USA)*

**Table 1a.** All sciences

| **China**  **USA** | **P** | **P(collab)** | **P(UIC)** | **%UIC** | **%Local** | **%Domestic** | **%Foreign** |
| --- | --- | --- | --- | --- | --- | --- | --- |
| **P** | 1 | .990^**^ | .932^**^ | .198 | -.068 | -.165 | .175 |
| **P(collab)** | .982^**^ | 1 | .925^**^ | .204 | -.053 | -.207 | .221^*^ |
| **P(UIC)** | .973^**^ | .954^**^ | 1 | .451^**^ | .042 | -.110 | .123 |
| **%UIC** | .279^**^ | .253^**^ | .434^**^ | 1 | .247^*^ | .120 | -.119 |
| **%Local** | .168^*^ | .188^*^ | .218^**^ | .206^**^ | 1 | .466^**^ | -.424^**^ |
| **%Domestic** | -.053 | -.091 | -.008 | .248^**^ | .270^**^ | 1 | -.981^**^ |
| **%Foreign** | .124 | .146 | .090 | -.166^*^ | -.276^**^ | -.909^**^ | 1 |

** Correlation is significant at the 0.01 level (2-tailed).

* Correlation is significant at the 0.05 level (2-tailed).

**Table 2a.** Life sciences

| **China**  **USA** | **P** | **P(collab)** | **P(UIC)** | **%UIC** | **%Local** | **%Domestic** | **%Foreign** |
| --- | --- | --- | --- | --- | --- | --- | --- |
| **P** | 1 | .988^**^ | .910^**^ | .039 | .037 | -.188 | .221 |
| **P(collab)** | .995^**^ | 1 | .922^**^ | .060 | .055 | -.195 | .228 |
| **P(UIC)** | .967^**^ | .971^**^ | 1 | .345^**^ | .123 | -.108 | .129 |
| **%UIC** | .243^**^ | .251^**^ | .406^**^ | 1 | .039 | .172 | -.186 |
| **%Local** | .109 | .129 | .174^*^ | .197^*^ | 1 | .537^**^ | -.513^**^ |
| **%Domestic** | -.123 | -.122 | -.066 | .189^*^ | .245^**^ | 1 | -.986^**^ |
| **%Foreign** | .137 | .138 | .101 | -.062 | -.150 | -.893^**^ | 1 |

** Correlation is significant at the 0.01 level (2-tailed).

* Correlation is significant at the 0.05 level (2-tailed).

**Table 3a.** Natural sciences

| **China**  **USA** | **P** | **P(collab)** | **P(UIC)** | **%UICs** | **%Local** | **%Domestic** | **%Foreign** |
| --- | --- | --- | --- | --- | --- | --- | --- |
| **P** | 1 | .987^**^ | .856^**^ | -0.001 | -.263^*^ | -0.168 | 0.173 |
| **P(collab)** | .994^**^ | 1 | .830^**^ | -0.009 | -.257^*^ | -0.204 | 0.208 |
| **P(UIC)** | .913^**^ | .900^**^ | 1 | .385^**^ | -0.111 | -0.093 | 0.104 |
| **%UICs** | 0.085 | 0.081 | .322^**^ | 1 | 0.149 | 0.087 | -0.081 |
| **%Local** | 0.039 | 0.046 | 0.109 | .256^**^ | 1 | .572^**^ | -.576^**^ |
| **%Domestic** | -0.038 | -0.042 | 0.036 | .203^*^ | .286^**^ | 1 | -.987^**^ |
| **%Foreign** | 0.036 | 0.039 | -0.039 | -.177^*^ | -.261^**^ | -.976^**^ | 1 |

** Correlation is significant at the 0.01 level (2-tailed).

* Correlation is significant a the 0.01 level (2-tailed).

*B. Universities included in the Linear Regression Analysis (SPSS) (China: 47, USA: 64).*

| **China** | **USA** |
| --- | --- |
| Beihang University | Arizona State Univ |
| Beijing Jiaotong University | Auburn Univ |
| Beijing University of Chemical Technology | Baylor Coll Med |
| Capital Medical University | Boston Univ |
| China Agricultural University | Brigham Young Univ |
| China Pharmaceutical University | Brown Univ |
| China University of Geosciences | Caltech |
| Chongqing University | Carnegie Mellon Univ |
| Dalian Polytechnic University | Case Western Reserve Univ |
| Donghua University | Clemson Univ |
| East China Normal University | Colorado State Univ |
| Fudan University | Columbia Univ |
| Fuzhou University | Cornell Univ |
| Harbin Institute of Technology | Dartmouth Coll |
| Huazhong University of Science and Technology | Drexel Univ |
| Hunan University | Duke Univ |
| Jiangsu University | E Carolina Univ |
| Jilin University | Emory Univ |
| Jinan University | Florida Intl Univ |
| Lanzhou University | Florida State Univ |
| Nanchang University | George Mason Univ |
| Nanjing Agricultural University | Georgetown Univ |
| Nanjing Medical University | Georgia Inst Technol |
| Nanjing University of Aeronautics and Astronautics | Harvard Univ |
| Nankai University | Icahn Sch Med - Mt Sinai |
| Northeast Normal University | Iowa State Univ |
| Northeastern University | Johns Hopkins Univ |
| Ocean University of China | Kansas State Univ |
| Shaanxi Normal University | Lehigh Univ |
| Shandong University | Louisiana State Univ |
| Shanghai Jiaotong University | Loyola Univ Chicago |
| Shanghai Normal University | Med Coll Wisconsin |
| Sichuan Normal University | Med Univ S Carolina |
| Soochow University | Michigan State Univ |
| South China Normal University | Mississippi State Univ |
| Southeast University | MIT |
| Southwest University | Montana State Univ |
| Tianjin University | N Carolina State Univ |
| Tongji University | North Dakota State Univ |
| Tsinghua University | Northeastern Univ - USA |
| University of Science and Technology Beijing | Northwestern Univ |
| Wuhan University | NYU |
| Wuhan University of Technology | Ohio State Univ |
| Xiangtan University | Ohio Univ |
| Xidian University | Oklahoma State Univ - Stillwater |
| Zhejiang Normal University | Oregon Hlth & Sci Univ |
| Zhejiang University of Technology | Oregon State Univ |
|  | Penn State Univ |
|  | Princeton Univ |
|  | Purdue Univ - Lafayette |
|  | Rensselaer Polytech Inst |
|  | Rice Univ |
|  | Rockefeller Univ |
|  | Rutgers State Univ - New Brunswick |
|  | San Diego State Univ |
|  | Southern Illinois Univ - Carbondale |
|  | Stanford Univ |
|  | Stony Brook Univ - SUNY |
|  | Temple Univ |
|  | Texas A&M Univ - College Stn |
|  | Texas Tech Univ |
|  | Thomas Jefferson Univ |
|  | Tufts Univ |
|  | Tulane Univ |
